# Sustained persistence of blockade antibodies against emerging GII.4 and GII.17 noroviruses revealed by a 5-year community-based serological study

**DOI:** 10.3389/fcimb.2026.1734113

**Published:** 2026-01-27

**Authors:** Dong-Jie Xie, Yu Zhang, Fei-Yuan Zhou, Mark Momoh Koroma, Zhi-Yan Liang, Xu-Fu Zhang, Ying-Chun Dai

**Affiliations:** 1 School of Traditional Chinese Medicine, Southern Medical University, Guangzhou, Guangdong, China; 2 The First Affiliated Hospital, and College of Clinical Medicine of Henan University of Science and Technology, Luoyang, China; 3 Department of Pharmacology and Toxicology, University of Toronto, Toronto ON, Canada; 4 Department of Epidemiology, Guangdong Provincial Key Laboratory of Tropical Disease Research, School of Public Health, Southern Medical University, Guangzhou, Guangdong, China; 5 Guangzhou Baiyun District Helong Street Community Healthcare Center, Guangzhou, Guangdong, China

**Keywords:** antibody persistence, blockade antibody, GII norovirus, HBGA, seroincidence, seroprevalence

## Abstract

**Introduction:**

GII noroviruses (NoVs) are the leading cause of viral gastroenteritis worldwide. A critical unresolved question is whether natural infection elicits long-lasting protective antibodies, which is paramount for guiding vaccine development. The long-term persistence of functional neutralizing antibodies against the predominant GII genogroup remains poorly characterized.

**Methods:**

In a 5-year community-based prospective cohort (2014-2018), we longitudinally assessed seroprevalence, seroincidence, and persistence of blockade antibodies against GII.4, GII.6, and GII.17 NoVs in 449 adults. HBGA (human histo-blood group antigen) associations with susceptibility were also analyzed.

**Results:**

GII.4 exhibited the highest and most stable seroprevalence (77.3-79.7%), while GII.17 seroprevalence rose sharply from 41.9% to 72.2%, capturing its emergence as an epidemic strain. Seroincidence was substantial, highest for GII.17 (30.4 per 100 person-years). In contrast to GI NoVs, GII.4 and GII.17 antibody exhibited exceptional persistence, with five-year persistence rate of 94.5% for GII.4 and 81.4% for GII.17, respectively, and no significant decay in blockade activity-both significantly exceeding the 61.2% persistence of GII.6. HBGA susceptibility patterns were distinct: GII.4 and GII.6 were associated with secretor status and Lewis antigens, while only blood type A was linked to GII.17 susceptibility.

**Conclusion:**

This study provides the first longitudinal evidence that natural infection with major GII NoVs, particularly GII.4 and the emerging GII.17, elicits robust and sustained functional antibody responses that persist for at least five years. This fundamental genogroup-specific disparity in immune durability, contrasting sharply with transient GI immunity, reveals a new dimension of NoVs immunology and provides a critical evidence base for the design of effective, long-lasting vaccines.

## Introduction

Noroviruses (NoVs) are the predominant cause of acute gastroenteritis (AGE) outbreaks worldwide, posing a substantial public health and economic burden. Globally, NoVs account for an estimated 699 million infections and over 200,000 deaths annually, with genogroup II (GII) strains responsible for the majority of these cases and pandemics (Liu et al., 2025; Lucero et al., 2021). Despite their predominance, critical gaps remain in understanding the serological features of herd immunity, particularly the persistence of functionally neutralizing antibodies and its implications for vaccine design against GII NoVs.

NoVs are classified into 10 genogroups (GI-GX), with GI, GII, and GIV primarily infecting humans (Hardy, 2005). Among these, GII NoVs, especially the GII.4 genotype, have dominated global outbreaks for decades, evolving through antigenic drift and shift to evade population immunity (Barclay et al., 2025; Zhang et al., 2024). Recent years have witnessed the emergence and global spread of non-GII.4 strains, including a significant GII.17 surge across Asia during 2014–2016 (De Graaf et al., 2015). The 2023/24 season marked a global resurgence of GII.17, with prevalence rising to 17–64% of GII detections in six European countries and the US. Therefore, our prospective cohort from 2014–2018 uniquely captured the precise serological footprint of this major epidemiological shift, providing an invaluable dataset to study the immune response to an emerging variant in real-time. While GII.4 declined, most strains clustered with Romania-2021 lineage, indicating a shift from the prior eight years of GII.4 Sydney[P16] dominance (Chhabra et al., 2024). Concurrently, GII.6 has been frequently identified as a major cause of AGE outbreaks in various countries, establishing itself as the second most common strain in young children (Parra et al., 2017). This continuous genetic diversification complicates prevention strategies and underscores the critical need for genotype-specific seroepidemiological data to inform surveillance and vaccine development.

Susceptibility to many NoVs is mediated by human histo-blood group antigens (HBGAs), which function as receptors or co-receptors, with infection risk influenced by polymorphisms in ABO, Lewis, and secretor status phenotypes (Nordgren and Svensson, 2019). While prior studies, including our longitudinal serosurveillance of GI NoVs, have elucidated HBGA-associated susceptibility and antibody dynamics for GI strains (Yu et al., 2023), similar insights for GII NoVs remain fragmented. For instance, GII.4 exhibits broad HBGA binding (Ayyar et al., 2025), whereas GII.17 and GII.6 demonstrate more distinct, strain-specific binding patterns (Huo et al., 2022; Zhang et al., 2015). These interactions directly impact population transmission patterns and are crucial for predicting vaccine efficacy, necessitating community-based studies to refine susceptibility profiles for predominant GII genotypes.

Serological surveillance is pivotal for capturing both symptomatic and asymptomatic infections, which are often substantially underestimated in stool-based studies (Thorne et al., 2018). Assays detecting blockade antibodies, which inhibit the binding of NoV viral-like particles (VLPs) or P proteins to HBGAs, serve as functional correlates of protective immunity. These antibodies are conformation- and genotype-dependent, offering superior specificity compared to cross-reactive IgG-based assays (Lindesmith et al., 2015; Yu et al., 2023). However, analogous longitudinal data for GII NoVs are critically scarce. The longevity of these functionally relevant blockade antibodies—a paramount factor for informing vaccination strategies and dosing intervals—remains largely unknown and represents a significant impediment to vaccine development. Our recent study on GI NoVs demonstrated genotype-dependent antibody persistence, with durations ranging from 2.3 to 4.8 years (Yu et al., 2023). This raises a pivotal question: does the dominant GII genogroup, with its distinct epidemiology, follow a similar pattern of waning immunity, or does it elicit a more robust and sustained antibody response? To address these gaps, we conducted a 5-year prospective cohort study in the Jidong community, China, to investigate the seroprevalence, seroincidence, and antibody persistence against the major GII NoVs genotypes GII.4, GII.6, and GII.17. We further analyzed HBGA-associated susceptibility patterns and antibody dynamics to provide a evidence base for vaccination strategies. This study directly complements our prior work on GI NoVs (Yu et al., 2023), providing a comprehensive serological framework for understanding GII NoV immunity and its persistence in a natural population.

## Materials and methods

### Study design and participants

This prospective cohort study, conducted from 2014 to 2018 in the Jidong community of northern China, utilized the same participant cohort and sampling framework as our previously published study on GI NoV. Briefly, from an initial pool of 10,043 adults (≥18 years), 456 participants were randomly selected for longitudinal follow-up. After exclusions for loss to follow-up, 449 individuals were included in the final analysis. The study design, ethical approvals, and informed consent procedures were identical to those previously described (Yu et al., 2023). Annual serum collections were conducted during the same seasonal window (Q4: October-December) each year from 2014 to 2018 to minimize confounding from seasonal variation in NoV transmission.

The minimal sample sizes for this study were calculated based on preliminary seroprevalence data specific to GII genotypes. Using the formula n = (Z_a_²) * P * (1 − P)/d² (where α = 0.05, Z_a_ = 1.96, and allowable error d = 0.15P), the required sample sizes were estimated to be 70 for GII.4 (P = 71%), 278 for GII.6 (P = 38%), and 286 for GII.17 (P = 37%). The final cohort size of 449 exceeds all these requirements.

### Sociodemographic data and biological samples

The procedures for collecting sociodemographic data (age, sex, education, ethnicity, marital status, and monthly income) via standardized questionnaires and the protocols for the annual collection (2014-2018) and storage (-80°C) of serum samples were performed as previously detailed (Yu et al., 2023). Saliva samples for HBGA phenotyping were collected in 2016 under the same conditions.

### HBGA phenotyping of saliva samples

The phenotypic characterization of ABO blood groups (A, B, O) and Lewis antigens (Le^a^, Le^b^, Le^x^, Le^y^) from saliva specimens was performed identically to our previous work (Yu et al., 2023), using established enzyme-linked immunosorbent assays (ELISAs) with specific monoclonal antibodies (Dai et al., 2017). The cutoff value (OD_450_ = 0.2) and quality control measures, including the use of pre-defined positive and negative controls on each plate, were consistent with the prior methodology.

### HBGA blockade assay

Consistent with established methodology for NoV serology, the HBGA-based blockade assay was employed as a functional surrogate for virus neutralization (Huang et al., 2005). The assay was performed as previously described, based on the principle of inhibiting the binding of viral P proteins to HBGA receptors present in saliva (Yu et al., 2023).

Briefly, saliva samples from selected donors, which demonstrated strong binding activity for the target genotypes, were diluted (1:1,000) and coated onto microplates. Subsequently, purified P proteins of GII.4, GII.6, or GII.17 were added at optimized concentrations of 5 µg/mL (Dai et al., 2017; Zhang et al., 2020). Genotype-specific, in-house-produced mouse antisera (diluted 1:3,000) were then used to detect the bound P proteins, followed by incubation with HRP-conjugated goat anti-mouse IgG (1:3,000) and development with TMB substrate.To assess the blockade activity of human serum antibodies, an additional pre-incubation step was introduced. Serum samples were mixed with the respective P proteins for 1 hour at 37°C prior to adding the mixture to the HBGA-coated plates. Control wells, containing P protein without serum pre-incubation, were included on each plate. The blocking index was calculated as: 100 − (OD sample/OD control) × 100. A sample was defined as positive for blockade antibodies if it demonstrated ≥50% inhibition. Each assay plate included well-characterized positive and negative serum controls for quality assurance (Yu et al., 2023).

### Statistical analysis

Seroprevalence and seroincidence rates were calculated using Pearson’s chi-square test with Fisher’s exact test. Bonferroni correction was used for the pairwise testing across years. Their 95% confidence intervals (95% CIs) were computed utilizing the Clopper-Pearson method of MedCalc software. Person-years for seroincidence were determined based on the time contributed by participants from seronegative to seropositive status. Associations between sociodemographic factors/HBGA phenotypes and seroprevalence were analyzed using generalized estimating equations (GEE) and multivariate logistic regression, respectively, adjusting for potential confounders (age, sex, ethnicity, marital status, education, income). The persistence and kinetics of blockade antibodies were modeled using a generalized linear model (GLM). Differences in antibody decay rates were assessed using empirical P-values from Fisher’s permutation test. All analyses were conducted using IBM SPSS Statistics v26.0 and Stata software, with a two-sided P-value < 0.05 considered statistically significant.

## Results

### Baseline characteristics of participants

A total of 449 adults from the Jidong community were included in this longitudinal analysis. The cohort had a mean age of 40.3 ± 12.7 years, with males comprising 55.5% of the participants. The vast majority were of Han Chinese ethnicity (97.1%) and married (88.9%). More than half (59.9%) had attained a college-level education or higher. In terms of household income, 54.3% reported a monthly income between ¥3001 and ¥5000 RMB (**Table 1**).

**Table 1:** Demographic Characteristics of the participants and Seroprevalence against GII NoV in Jidong community-based cohort in 2014.

Characteristics	No. of test	GII.4		GII.6		GII.17	
		Positive (%)	P value	Positive (%)	P value	Positive (%)	P value
**Age group, years**			0.836		0.717		**0.044**
18-29	132	104(78.79)		46(34.85)		52(39.40)	
30-39	108	82(75.93)		37(34.26)		34(31.50)	
40-49	91	67(73.63)		39(42.86)		45(49.45)	
50-59	77	62(80.52)		29(37.66)		39(59.65)	
60-80	41	32(78.05)		14(34.15)		18(43.90)	
**Sex**			0.018^*^		0.767		0.083
Male	249	182(73.09)		90(36.14)		120(55.00)	
Female	200	165(82.50)		75(37.50)		80(45.00)	
**Ethnic group**			0.329		0.897		0.163
Han	436	335(76.83)		160(36.70)		185(42.43)	
ELSE	13	12(92.31)		5(38.46)		3(23.08)	
**Marital status**			0.562		0.139		0.831
Unmarried	38	32(84.21)		9(23.68)		16(42.11)	
Divorced or widowed	12	9(75.00)		3(25.00)		4(33.33)	
Married	399	306(76.69)		153(38.35)		168(42.11)	
**Education**			0.651		0.132		**<0.001**
Illiteracy or primary school	13	10(76.92)		7(53.85)		5(38.46)	
Junior high school	167	133(79.64)		53(31.74)		74(44.31)	
College or higher	269	204(76.12)		105(39.03)		188(69.89)	
**Income**			0.452		0.514		0.912
≤ ¥3,000	165	132(80.00)		65(39.39)		69(41.82)	
¥3,001-5,000	244	183(75.00)		88(36.07)		101(41.39)	
> ¥5000	40	32(80.00)		12(30.00)		18(45.00)	

*P < 0.05, **P < 0.01. The bold values indicate statistical signiﬁcance.

### Seroprevalence and annual variation of GII NoV blockade antibodies

**Figure 1:**
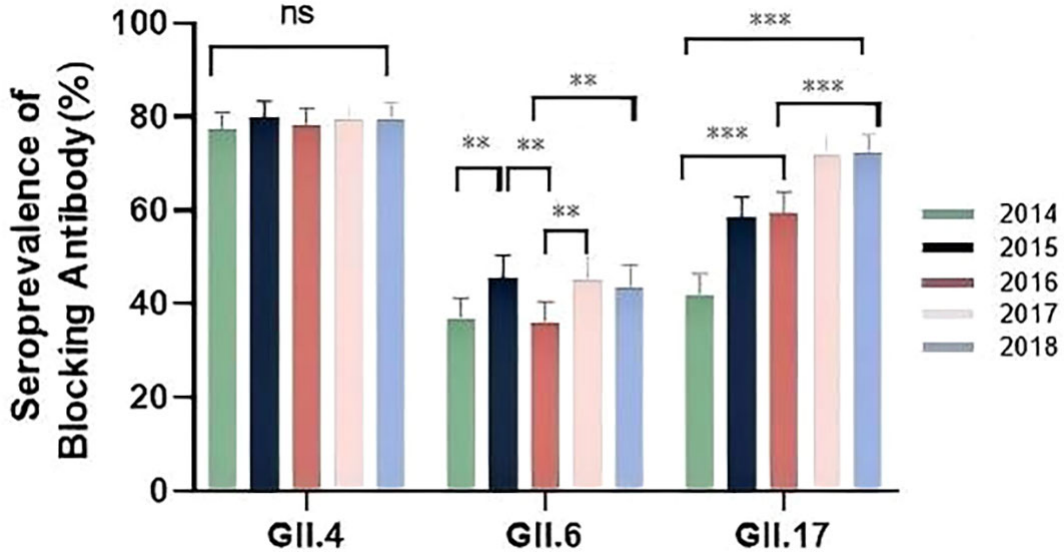
Seroprevalence of anti-GII blockade antibodies in 2014-2018. Serum samples from 449 participants were tested annually. The y-axis indicates seroprevalence. Statistically significant differences in seroprevalence between years were assessed for each genotype using Pearson’s chi-square test with Fisher’s exact test. Bonferroni correction was used for the pairwise testing across years. Significance levels are indicated as follows: **P < 0.01; ***P < 0.001; NS, not significant. For GII.6, seroprevalence was significantly higher in 2015 (45.7%), 2017 (45.0%), and 2018 (43.7%) compared to 2014 (36.8%) and 2016 (36.1%). For GII.17, seroprevalence increased significantly over time: the rate in 2014 (41.9%) was lower than in 2015 (58.4%) and 2016 (59.5%), which were in turn lower than in 2017 (71.7%) and 2018 (72.2%). No significant interannual differences were observed for GII.4 seroprevalence. Error bars represent 95% confidence intervals.

From 2014 to 2018, we annually assessed the seroprevalence of blockade antibodies against GII.4, GII.6, and GII.17 NoVs. GII.4 exhibited the highest and most stable seroprevalence, starting at 77.3% in 2014 and remaining between 78.2% and 79.7% in subsequent years (P = 0.867, **Figure 1**). In contrast, GII.17 demonstrated significant temporal variation (P = 0.001), with seroprevalence rising sharply from a low of 41.9% in 2014 to a high of 72.2% in 2018 (**Figure 1**), coinciding with its emergence as an epidemic strain in Asia during this period (De Graaf et al., 2015; Fu et al., 2015). GII.6 had the lowest overall seroprevalence, which increased significantly over the study period from 36.1% in 2014 to 45.7% in 2018 (P = 0.003, **Figure 1**).

### Sociodemographic factors associated with seroprevalence

Analysis of sociodemographic variables revealed that males had a significantly lower seroprevalence of GII.4 NoV at baseline in 2014 (P = 0.018, **Table 1**), a trend that was sustained in the longitudinal analysis (**Table 2**). No significant associations were found between sociodemographic factors and GII.6 seroprevalence. For GII.17, higher seroprevalence was associated with older age. At baseline, older age groups showed a significant correlation (P = 0.044, **Table 1**). Longitudinal analysis confirmed that individuals aged 50-59 (AOR = 2.60, 95% CI: 1.53-4.43) and 60-80 (AOR = 2.56, 95% CI: 1.40-4.69) had significantly higher seroprevalence compared to younger adults (**Table 2**).

**Table 2:** Factors associated with the seroprevalence of GII NoVs in Jidong community-based cohort, 2014-2018.

Characteristics	GII.4		GII.6		GII.17	
	OR (95% CI)^a^	Adj-OR (95% CI)	OR (95% CI)	Adj-OR (95% CI)	OR (95% CI)	Adj-OR (95% CI)
Age group, years						
18-29	1.00(Ref)	1.00(Ref)	1.00(Ref)	1.00(Ref)	1.00(Ref)	1.00(Ref)
30-39	0.90(0.50-1.60)	0.88(0.55-1.43)	0.92(0.60-1.41)	1.12(0.77-1.63)	0.75(0.51-1.10)	0.91(0.59-1.41)
40-49	0.72(0.40-1.30)	0.65(0.03-1.25)	1.01(0.60-1.58)	0.73(1.09-1.80)	1.35(0.87-2.09)	1.57(0.95-2.59)
50-59	1.25(0.63-2.46)	1.09(0.50-2.37)	1.02(0.64-1.63)	1.02(0.58-1.81)	1.36(0.86-2.15)	**2.60(1.53-4.43)**
60-80	0.97(0.42-2.23)	0.93(0.40-2.19)	0.81(0.43-1.49)	1.09(0.56-2.11)	1.24(0.71-2.16)	**2.56(1.40-4.69)**
Sex						
Male	1.00(Ref)	1.00(Ref)	1.00(Ref)	1.00(Ref)	1.00(Ref)	1.00(Ref)
Female	**1.95(1.25-3.05)**	**2.01(1.26-3.181)**	1.11(0.81-1.52)	0.88(0.63-1.22)	0.96(0.71-1.29)	1.03(0.76-1.41)
Ethnic group						
Han	1.00(Ref)	1.00(Ref)	1.00(Ref)	1.00(Ref)	1.00(Ref)	1.00(Ref)
ELSE	2.68(0.47-15.36)	2.39(0.40-14.14)	1.90(0.80-4.52)	1.85(0.82-4.17)	0.97(0.47-2.00)	1.05(0.20-2.21)
Marital status						
Unmarried	1.00(Ref)	1.00(Ref)	1.00(Ref)	1.00(Ref)	1.00(Ref)	1.00(Ref)
Divorced or widowed	0.94(0.22-4.04)	1.40(0.30-6.54	1.49(0.56-3.95)	1.20(0.38-3.78)	1.24(0.52-2.97)	1.38(0.60-3.17)
Married	0.64(0.19-2.18)	0.88(0.26-2.99)	0.88(0.30-2.62)	1.75(0.96-3.21)	1.73(0.66-4.57)	2.19(0.80-6.00)
Education						
Illiteracy or primary school	1.00(Ref)	1.00(Ref)	1.00(Ref)	1.00(Ref)	1.00(Ref)	1.00(Ref)
Junior high school	0.94(0.26-3.45)	0.99(0.26-3.78)	0.77(0.55-1.07)	0.57(0.19-1.68)	1.88(0.68-3.97)	2.23(0.85-5.85)
College or higher	0.80(0.22-2.86)	0.99(0.24-4.08)	0.67(0.37-1.19)	0.74(0.24-2.35)	1.88(0.76-4.61)	2.59(0.92-7.27)
Income						
≤ ¥3,000	1.00(Ref)	1.00(Ref)	1.00(Ref)	1.00(Ref)	1.00(Ref)	1.00(Ref)
¥3,001-5,000	0.73(0.46-1.15)	0.77(0.37-2.09)	0.77(0.55-1.07)	0.71(0.50-1.02)	0.97(0.70-1.32)	1.06(0.74-1.52)
> ¥5000	0.82(0.36-1.87)	0.88(0.47-1.28)	0.67(0.37-1.19)	0.65(0.35-1.19)	1.35(0.78-2.35)	1.42(0.81-2.47)

a OR, odds ratio; 95% CI, 95% conﬁdence interval. The bold values indicate statistical signiﬁcance.

### Seroincidence rates and associated risk factors

**Figure 2:**
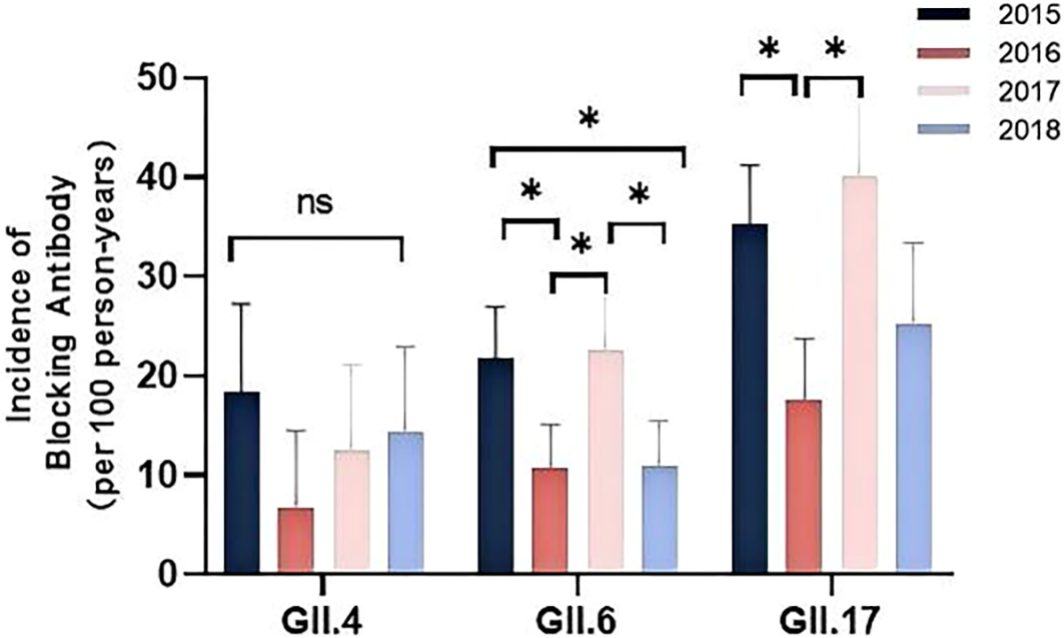
Seroincidence of anti-GII blockade antibodies in 2014-2018. Anti-GII.4, GII.6, and GII.17 antibodies were tested annually in 449 participants. The y-axis indicated the seroincidence. The colored bars indicated the corresponding seroincidence rates for different years. Error bars indicated a 95% confidence interval. *P < 0.05; NS, nonsignificant.

Seroincidence, defined as the rate of new seroconversions (from negative to positive for blockade antibodies) per 100 person-years of observation, was calculated. Over the four-year follow-up, the seroincidence rates were 13.2 per 100 person-years for GII.4, 17.0 for GII.6, and 30.4 for GII.17. The seroincidence of GII.4 remained stable across all years (**Figure 2**). Conversely, both GII.6 and GII.17 showed significant yearly fluctuations, with notably higher peaks in 2015 and 2017 (P < 0.01, **Figure 2**).

Several demographic factors were linked to seroincidence: For GII.4, incidence was significantly higher among females (P = 0.028) and individuals who were divorced or widowed (P = 0.02, **Table 3**). For GII.6, higher incidence was observed in participants aged 30–39 years (P < 0.001), those of non-Han Chinese ethnicity (P = 0.008), and individuals with a monthly income below ¥3000 (P = 0.023, **Table 3**). For GII.17, educational attainment was a significant factor, with individuals having at least a junior high school education showing a higher incidence than those with primary education or less (P = 0.042, **Table 3**).

**Table 3:** Factors associated with seroincidence against GII NoV in Jidong community-based cohort in 2014-2018.

Characteristics	GII.4			GII.6			GII.17		
	No. of person-years	Seroincidence (per 100 person-years)^a^	P value	No. of person-years	Seroincidence (per 100 person- years)	P value	No. of person-years	Seroincidence (per 100 person-years)	P value
**Age group, years**			0.868			**0.003**			0.692
18-29	72	8(11.11)		263	29(11.02)		234	77(32.91)	
30-39	115	18(15.65)		286	68(23.78)		221	64(28.96)	
40-49	98	11(11.22)		224	35(15.63)		129	37(28.68)	
50-59	49	7(14.29)		171	29(16.96)		111	30(27.03)	
60-80	45	6(13.33)		118	19(16.10)		62	22(35.48)	
**Sex**			**0.028**			0.272			0.154
Male	256	27(10.55)		579	102(17.62)		413	124(30.02)	
Female	123	23(18.70)		483	78(16.15)		344	120(34.88)	
**Ethnic group**			0.509			**0.042**			0.101
Han	374	49(13.10)		1039	172(16.55)		735	215(29.25)	
ELSE	5	1(20.00)		23	8(34.78)		22	10(45.45)	
**Marital status**			**0.002**			0.796			0.100
Unmarried	24	3(12.50)		107	21(19.63)		24	6(25.00)	
Divorced or widowed	7	4(57.14)		34	5(14.71)		56	27(48.21)	
Married	348	43(12.36)		921	154(16.72)		677	197(29.10)	
**Education**			0.187			0.553			**0.042**
Illiteracy or primary school	9	3(33.33)		24	3(12.50)		27	6(22.22)	
Junior high school	131	16(12.21)		414	66(15.94)		261	77(29.50)	
College or higher	239	31(12.97)		624	111(17.79)		469	147(31.34)	
**Income**			0.214			**0.024**			0.518
≤ ¥3,000	118	20(16.95)		361	77(21.33)		277	83(39.96)	
¥3,001-5,000	228	28(12.28)		600	87(14.50)		423	143(33.81)	
> ¥5000	33	2(6.05)		101	16(15.84)		57	20(35.09)	

a The new infection of GII NoV was defined as the seroconversion from negative to positive for the corresponding genotype. The seroincidence of GII NoV was determined by dividing the number of newly infected individuals by the person-years observed during the study period from 2014 to 2018. The bold values indicate statistical signiﬁcance.

### HBGA phenotypes and susceptibility to GII NoV infection

The distribution of HBGA phenotypes among participants was as follows: ABO phenotypes A (31.2%), B (18.0%), AB (19.4%), O^+^ (18.5%), and O^-^ (12.9%); Lewis phenotypes Le^b+^/Le^y+^ (56.1%), Le^a+b+^/Le^x+y+^ (31.0%), and Le^a+^/Le^x+^ (12.9%). The majority (87.1%) were secretors.

**Figure 3:**
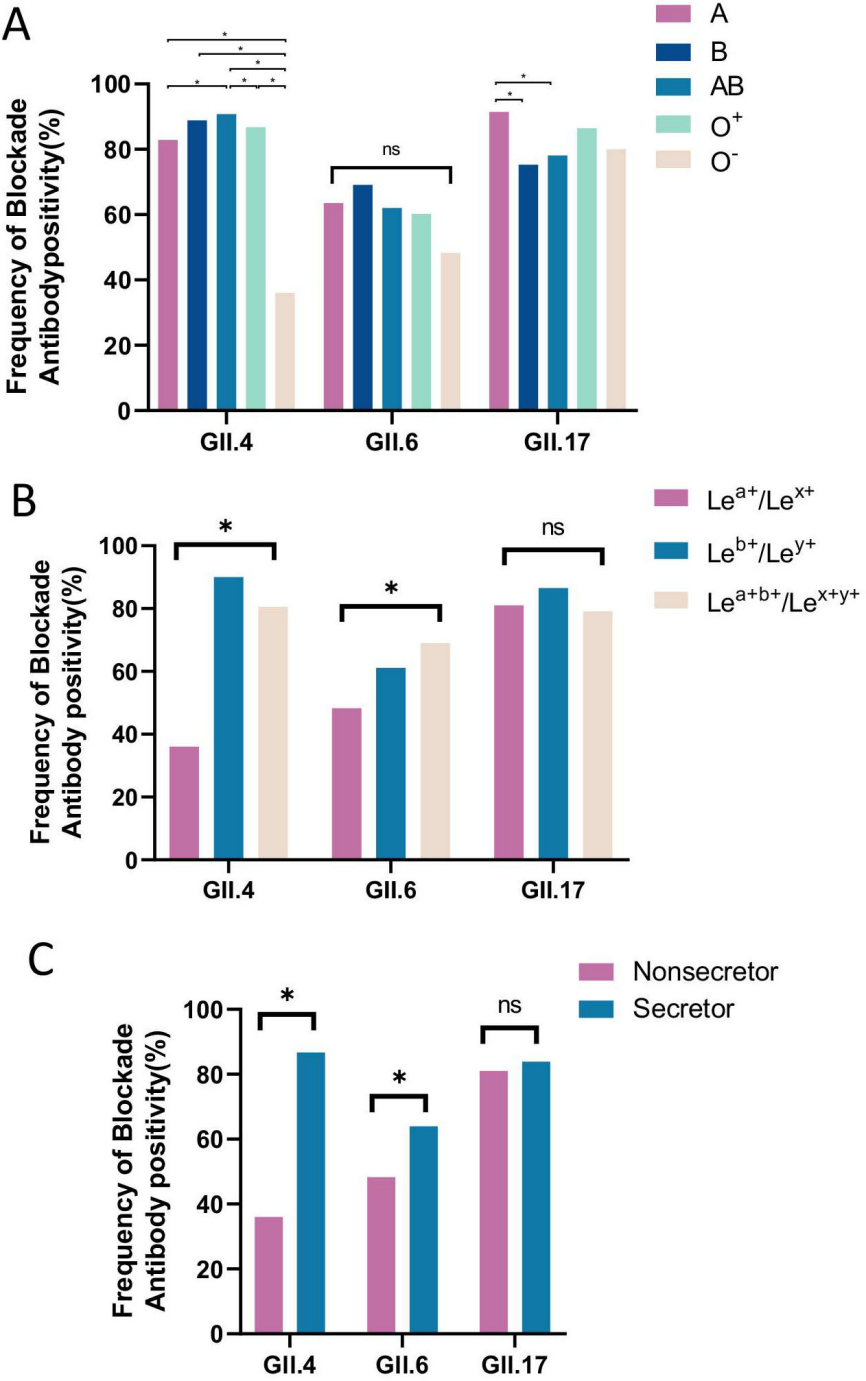
Blockade antibody positive rates of GII NoVs for participants with different ABO blood types **(A)**, Lewis phenotypes **(B)**, and secretor status **(C)**. The y-axis indicated the frequency of blockade antibodies. Different colored bars showed different human histo-blood group antigen (HBGA) phenotypes. *P < 0.05; NS, nonsignificant.

HBGA phenotype distributions differed significantly between blockade antibody-positive and negative groups (**Figure 3**, **Table 4**). Individuals with the O^-^ phenotype had a markedly lower positive rate for GII.4 antibodies (32.7%) compared to all other ABO phenotypes (82.9-94.3%, P<0.001). Conversely, those with blood type A showed the highest positive rate for GII.17 antibodies (91.4%, **Table 4**). Notably, the associations between ABO blood types and susceptibility were strongest and most complex for GII.4, involving multiple phenotypes, whereas for GII.17, susceptibility was primarily and more simply linked to blood type A.Similarly, positive rates were significantly higher for individuals with Lewis phenotypes Le^x+^/Le^y+^ (GII.4: 90.5%, GII.6: 61.1%) and Le^a+b+^/Le^x+y+^ (GII.4: 81.3%, GII.6: 69.1%) than for the Le^a+^/Le^x+^ phenotype (GII.4: 32.5%, GII.6:48.3%). Secretors were also at consistently higher risk across genotypes (**Figure 3**, **Table 4**).

**Table 4:** Association between different HBGAs phenotypes and susceptibility to GII NoVs using logistic regression analysis in Jidong community-based cohort, 2014-2018.

Status	Total (n=449)	GII.4			GII.6				GII.17	
		Positive (%)	OR (95%CI)^a^	P value	Positive (%)	OR (95%CI)	P value	Positive (%)	OR (95%CI)	P value
Blood type										
A	140	116(82.86)	9.92(4.91-20.03)	**<0.001**	89(63.57)	1.88(1.01-3.54)	**0.047**	128(91.43)	2.67(1.12-6.34)	**0.026**
B	81	72(88.89)	16.42(6.79-39.74)	**<0.001**	56(69.14)	2.34(1.15-4.74)	**0.019**	61(75.31)	0.76(0.34-1.71)	0.511
AB	87	82(94.25)	33.66(11.71-86.81)	**<0.001**	54(62.07)	1.91(0.96-3.81)	0.066	68(78.16)	0.90(0.40-2.02)	0.788
O^+^	81	71(85.54)	12.15(5.34-27.62)	**<0.001**	51(61.45)	1.74(0.87-3.48)	0.119	70(86.42)	1.59(0.65-3.90)	0.310
O^-^	60	19(32.76)	1.00(Ref)		28(48.28)	1.00(Ref)		48(80.00)	1.00(Ref)	
Lewis status										
Le^a+^/Le^x+^	58	19(32.54)	1.00(Ref)		28(48.28)	1.00(Ref)		47(81.03)	1.00(Ref)	
Le^b+^/Le^y+^	252	228(90.48)	19.50(9.77-38.92)	**<0.001**	154(61.11)	1.65(0.91-3.00)	0.099	218(86.51)	1.13(0.52-2.44)	0.763
Le^a+b+^/Le^x+y+^	139	113(81.29)	8.92(4.45-17.87)	**<0.001**	96(69.06)	2.55(1.33-4.88)	**0.005**	110(79.14)	1.61(0.98-29.18)	0.059
Secretor status										
Nonsecretor	58	19(32.54)	1.00(Ref)		28(48.28)	1.00(Ref)		47(81.03)	1.00(Ref)	
Secretor	391	341(87.21)	14.00(7.50-26.12)	**<0.001**	250(63.94)	1.93(1.09-3.42)	**0.025**	328(83.89)	0.82(0.40-1.67)	0.585

a OR: Odds ratio, 95% CI: 95% confidence interval; The bold values indicate statistical signiﬁcance.

Multivariate logistic regression confirmed these associations. Compared to the O^-^ reference group, phenotypes A (GII.4: OR = 9.92; GII.6: OR = 1.88; GII.17: OR = 2.67), B (GII.4: OR = 16.42; GII.6: OR = 2.34), AB (GII.4: OR = 33.66), and O^+^ (GII.4: OR = 12.15) showed elevated susceptibility. The Le^a+b+^/Le^x+y+^ phenotype was associated with a significantly higher risk of infection for both GII.4 (OR = 7.31, P < 0.001) and GII.6 (OR = 2.55, P < 0.005). Secretor status significantly increased the risk for GII.4 (OR = 11.49, P < 0.001) and GII.6 (OR = 1.93, P = 0.025) infections (**Table 4**).

### Persistence and kinetics of blockade antibodies

**Figure 4:**
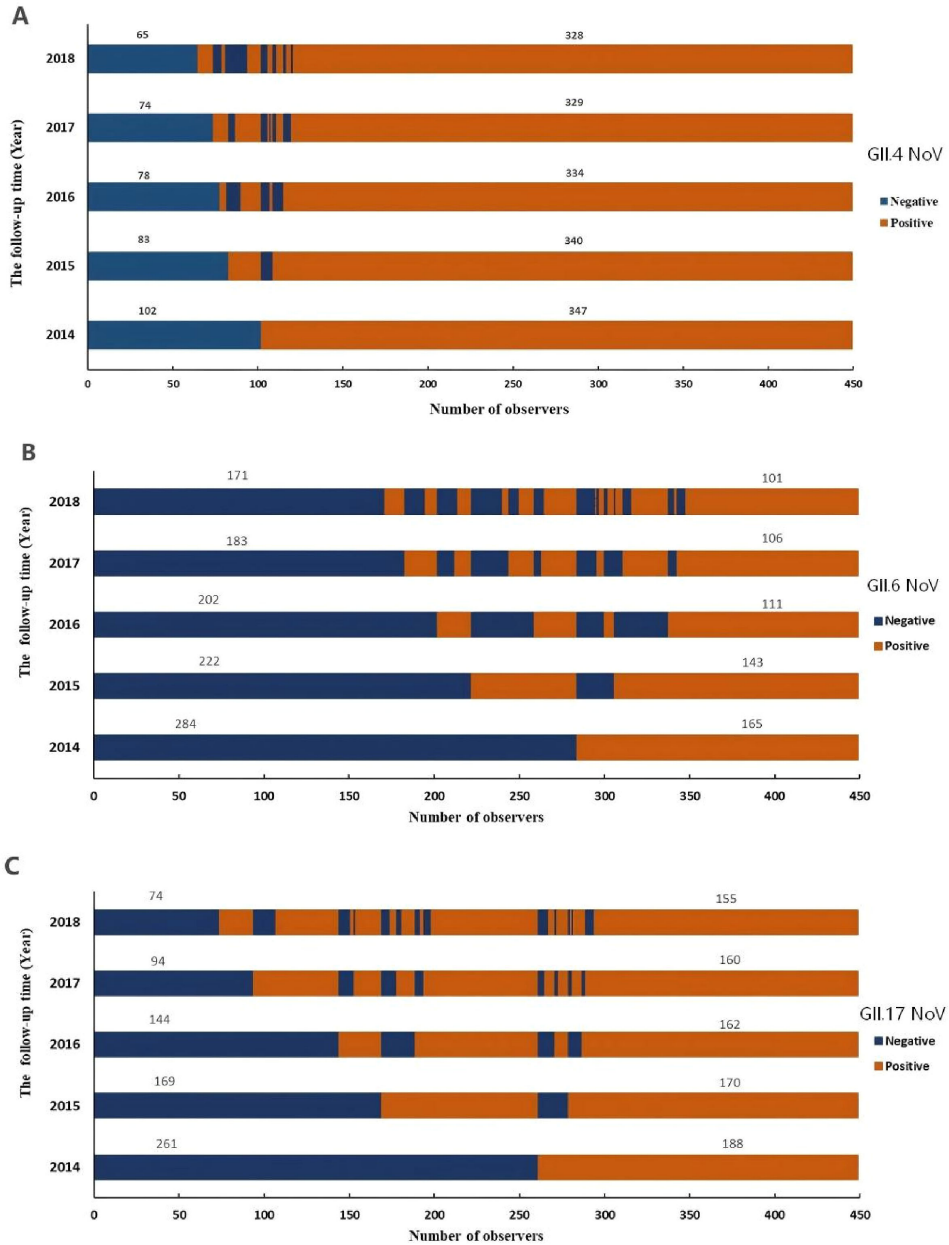
Persistence and Kinetics of blockade antibody against GII.4 **(A)**, GII.6 **(B)**, and GII.17 **(C)** NoV from 2014 to 2018. The y-axis indicated the follow-up year, and the x-axis illustrated the number of observations. Blue indicated the number of participants with antibody positive, whereas orange indicated those with antibody negative. The number above the blue bar indicated individuals with antibody persistent positive, and the number above the orange bar indicated individuals with antibody persistent negative.

Antibody persistence was analyzed in individuals seropositive at baseline (2014): 347 for GII.4, 165 for GII.6, and 188 for GII.17 (**Figure 4**). The five-year antibody persistence rate was highest for GII.4 at 94.52% (328/347), followed by GII.17 at 81.45% (155/188), and GII.6 at 61.21% (101/165) (**Figures 4A-C**).

The blockade ability of these antibodies remained stable over time. No statistically significant decline in blockade activity was observed from 2014 to 2018 for any of the three genotypes (GII.4: P = 0.715; GII.6: P = 0.787; GII.17: P = 0.124; **Table 5**).

**Table 5:** Blocking rate of blockade antibodies against GII NoV, 2014-2018.

Observation time (year)	GII.4		GII.6		GII.17	
	Blocking rate (%) (Mean ± S)	P value	Blocking rate (%) (Mean ± S)	P value	Blocking rate (%) (Mean ± S)	P value
2014	85.7 ± 10.8	0.715	65.1 ± 13.0	0.787	61.9 ± 11.7	0.124
2015	86.0 ± 11.8		66.0 ± 13.4		65.9 ± 13.9	
2016	85.5 ± 12.1		63.3 ± 13.1		64.4 ± 13.0	
2017	85.5 ± 11.9		65.4 ± 14.5		64.8 ± 12.3	
2018	85.5 ± 11.1		64.9 ± 13.8		65.0 ± 13.3	

seroreversion rates for GII.4 (range: 2.1%-4.2%) and GII.17 (range: 6.7%-10.7%) were low and stable from 2015 to 2018, with no significant interannual differences (P = 0.269 and P = 0.445, respectively; **Table 6**). In stark contrast, the seroreversion rate for GII.6 was significantly higher in 2016 (33.66%) compared to other years (P < 0.001, **Table 6**).

**Table 6:** Seroreversion rates of blockade antibodies against GII NoV in Jidong community-based cohort in 2014-2018.

Observation time (year)		GII.4			GII.6			GII.17	
	No. of positive	No. of Seroreversion (%)	P value	No. of positive	No. of Seroreversion (%)	P value	No. of positive	No. of Seroreversion (%)	P value
2015	347	7 (2.08)	0.213	165	22 (13.33)	**<0.001**	188	18 (9.57)	0.445
2016	359	13 (3.62)		202	**69 (33.66)**		262	28 (10.69)	
2017	352	8 (2.27)		162	25 (15.43)		267	18 (6.74)	
2018	339	15 (4.18)		202	33 (12.38)		322	30 (9.32)	

The bold values indicate statistical significance.

## Discussion

The most significant finding of this 5-year community-based longitudinal study is the exceptional persistence of blockade antibodies against GII.4 and GII.17 NoVs, which stands in direct and stark contrast to the significant antibody decay we previously observed for GI NoVs in the same cohort (Yu et al., 2023). This genogroup-specific disparity in immune memory is a pivotal discovery, suggesting fundamental differences in the nature of immunity across genogroups, with profound implications for vaccine development and surveillance strategies.

The high, stable seroprevalence of GII.4 (77.3-79.7%) reflects its persistent community circulation and enduring dominance as the primary driver of global pandemics (Barclay et al., 2025; Van Beek et al., 2018; Wei et al., 2021). In contrast, GII.17 seroprevalence rose sharply from 41.9% to 72.2% between 2014 and 2018, capturing the serological footprint of its emergence as a major epidemic variant across Asia during this period (Chan et al., 2015; Fu et al., 2015; Matsushima et al., 2015). The seroincidence rates further underscore a substantial burden of infection, particularly for GII.17 (30.38 per 100 person-years), greatly exceeding estimates from stool-based surveillance (Chhabra et al., 2024; Gomes et al., 2025). This discrepancy highlights the significant proportion of asymptomatic or unreported infections detectable only through longitudinal serological studies, confirming the true burden of GII NoV infection is markedly underestimated.

Most strikingly, antibody kinetics differed dramatically between genogroups. In our prior study, GI antibodies exhibited significant decay, with estimated durations of 2.3 to 4.8 years and annual decay rates ranging from -3.6% to -5.9% (Yu et al., 2023). In direct contrast, naturally acquired GII antibodies, particularly against GII.4 and GII.17, were maintained without significant decay over the same five-year period in the same cohort.

The vast majority of GII.4-positive individuals (94.5%) maintained seropositivity over five years, with no significant decline in blockade activity. Similarly, 81.5% of GII.17-positive individuals remained seropositive. This sustained antibody response suggests that natural infection with these GII genotypes is associated with a more sustained humoral immune response compared to GI infections. We hypothesize that the sustained antibody levels could be driven by fundamental differences in immunodominant epitopes that confer greater stability to the humoral response, or by more frequent subclinical re-exposure to continually circulating GII.4 and emergent GII.17 variants, providing natural boosting.

The significantly lower persistence rate and the anomalously high seroreversion observed for GII.6 in 2016 present a intriguing contrast to the stability of GII.4 and GII.17 antibodies. This may indicate a fundamentally different, perhaps less robust, immune response elicited by natural GII.6 infection compared to GII.4 and GII.17 (Kakakhel et al., 2022; Parra et al., 2017). This genotype-specific disparity warrants further investigation through concurrent genetic sequencing of circulating strains to directly link antigenic variation with serological outcomes.

HBGA phenotypes again confirmed their role as critical determinants of host susceptibility, but with patterns distinct from those we observed for GI NoVs. For GII.4 and GII.6, secretor status and specific Lewis phenotypes (Le^b+^/Le^y+^ and Le^a+b+^/Le^x+y+^) were strongly associated with infection risk, consistent with their broad HBGA binding profiles (Dai et al., 2015; Zhang et al., 2020). Notably, individuals with the O^-^ phenotype were significantly protected against GII.4, a finding that aligns with earlier reports but differs from the susceptibility of O^+^ individuals we observed for GI.2 and GI.3 (Yu et al., 2023). For GII.17, blood type A was the primary susceptibility factor, which correlates with its known binding preference for A and B antigens (Tarris et al., 2022; Zhang et al., 2015). This is a simpler susceptibility profile compared to the multi-faceted HBGA associations seen with GI and other GII types. These divergent HBGA binding patterns between GI and GII genotypes underscore the necessity for broadly protective, multivalent vaccines that can overcome the complex host genetic restrictions to infection.

The epidemiological patterns also revealed genotype-specific and genogroup-specific differences. The higher seroprevalence and seroincidence of GII.4 in females suggest potential gender-based differences in exposure or immune response, a trend not observed in our GI study. Furthermore, the increased risk associated with non-Han ethnicity and lower income for GII.6 points to potential socioeconomic and cultural disparities in exposure, factors that should be considered in targeted public health interventions. The observed sociodemographic associations likely reflect complex interactions between genotype-specific transmission patterns, host behavior, and immune history. For instance, the higher GII.4 seroprevalence and seroincidence in females may be related to caregiving roles or occupational exposure, a hypothesis that warrants future study. The increased GII.6 risk associated with non-Han ethnicity and lower income may point to socioeconomic disparities in living conditions, access to sanitation, or dietary practices influencing exposure. The lack of a uniform pattern across genotypes underscores the distinct epidemiology of each strain and highlights the need for multifactorial models in understanding NoV transmission dynamics.

Our findings on the sustained persistence of GII.4 and GII.17 blockade antibodies align with and significantly extend a growing body of evidence on the robustness of GII immune responses. A recent multi-year study also reported remarkably stable blockade antibody titers against historical and contemporary GII.4 variants, suggesting a long-lasting immune imprinting effect from repeated exposures (Lindesmith et al., 2022; Su et al., 2023). Furthermore, the strong association between secretor status and susceptibility to GII.4 and GII.6 infection that we observed has been consistently demonstrated in both outbreak investigations and challenge studies, reinforcing the critical role of HBGAs as a genetic determinant of risk (Dai et al., 2017; Zhang et al., 2020). Our longitudinal community-based data provide a crucial nuance: while HBGA phenotype dictates initial susceptibility, infection can elicit a functional antibody response that is sustained for years. This is particularly relevant for GII.17: while our finding that susceptibility is conferred primarily by blood type A aligns with its known restricted binding profile (Chhabra et al., 2024; Jing et al., 2025), the observation that infection elicits sustained functional immunity provides a novel, key insight for predicting population immunity against this re-emerging genotype. Collectively, these recent studies, combined with our longitudinal evidence, paint a picture of GII NoVs as pathogens that, despite their diversity, can induce potent and lasting functional immunity upon infection, a cornerstone consideration for designing vaccines that aim to mimic natural immunity.

While the samples for this study were collected between 2014 and 2018, the fundamental immunological insights we provide are timeless and critically address a core question in the field. The recent global resurgence of GII.17 in the 2023/24 season (Chhabra et al., 2024; Barclay and Vinjé, 2025) underscores the persistent threat posed by this genotype and validates the importance of understanding the immune response it elicits. Our data, captured during its initial emergence, provide the first longitudinal evidence that infection with this re-emerging variant induces durable, long-lasting functional antibodies. This finding is crucial for predicting population susceptibility and immunity against current and future GII.17 waves. Similarly, the sustained circulation and evolution of GII.4 variants necessitates a deep understanding of the long-term immune response they elicit, which our study definitively shows is robust and persistent. Thus, rather than diminishing its relevance, the specific timeframe of our cohort uniquely allowed us to capture the natural history of an emerging variant and establish a foundational principle of GII antibody durability that is directly applicable to the current NoV landscape.

This study has several limitations. The exclusion of children under 18 limits insights into a highly vulnerable population. The focus on a single community in Northern China necessitates caution in generalizing the findings to other regions with different demographic and genetic backgrounds. Furthermore, as a community-based study in an area of endemic NoV circulation, we cannot definitively determine whether the sustained antibody levels observed, particularly for GII.4, are due to a single infection eliciting a intrinsically long-lived response or to repeated re-infection providing natural boosting. Our data demonstrate the functional maintenance of antibodies over five years but do not delineate the underlying mechanism. Future studies incorporating frequent sampling and molecular surveillance to link re-exposure events with antibody kinetics would be valuable.

## Conclusion

In conclusion, this study not only documents the high community burden of GII NoVs but fundamentally advances our understanding of NoVs immunity by demonstrating a clear genogroup-specific disparity in antibody persistence. The exceptional durability of GII.4 and GII.17 blockade antibodies provides a mechanistic basis for the long-term immune imprinting observed in populations and strongly supports the feasibility of developing vaccines that elicit sustained protection. Future vaccine efforts must account for these fundamental differences in immune durability between GI and GII genogroups to achieve broad and long-lasting efficacy.

## Data Availability

The original contributions presented in the study are included in the article/supplementary material. Further inquiries can be directed to the corresponding authors.
